# Distribution patterns and environmental correlates of water mites (Hydrachnidia, Acari) in peatland microhabitats

**DOI:** 10.1007/s10493-013-9692-8

**Published:** 2013-04-06

**Authors:** Mariusz Więcek, Peter Martin, Maciej Gąbka

**Affiliations:** 1 Department of Animal Morphology, Faculty of Biology, Adam Mickiewicz University, Umultowska 89, 61-614 Poznań, Poland; 2 Department of Limnology, Zoological Institute, Christian-Albrechts-Universität zu Kiel, Olshausenstr. 40, 24098 Kiel, Germany; 3 Department of Hydrobiolology, Faculty of Biology, Adam Mickiewicz University, Umultowska 89, 61-614 Poznań, Poland

**Keywords:** Peatlands, Spring fen, Habitat, *Sphagnum*, Poor-rich gradient, Vegetation, Acidification

## Abstract

In Europe peatlands are wetlands of postglacial origin. Because of climatic changes and agricultural activities (i.e. drainage and peat extraction), they are one of the most endangered ecosystems worldwide. Water mites are well known as indicators of changing environments in other ecosystems such as springs and lakes. For our study we selected seven peatlands located in North-Western Poland and focused on water mite distribution and associated habitat and water quality variables. We described water mite fauna in various microhabitats (aquatic and semiaquatic) along the mineral-richness gradient to test whether this gradient is reflected in the composition of water mite assemblages. We selected conductivity, pH and vegetation as variables reflecting the poor-rich gradient. Additionally, we measured water depth, temperature and dissolved oxygen, which are often important parameters for water mites. We also noted presence of prey and host taxa of particular water mite species. Based on physicochemical parameters we identified three types of habitats harbouring three distinctive species groups of water mites. We were able to distinguish species that appear to be typical of spring fens (e.g. *Hygrobates norvegicus*, *Lebertia separata*), connected with acidic, nutrient poor pools (e.g. *Arrenurus neumani*, *A. pustulator*) and species seemingly typical of temporary habitats dominated by *Sphagnum* mosses (e.g. *Piersigia intermedia*, *Zschokkea oblonga*, *A. stecki*). The poor-rich gradient is strongly reflected in the composition of water mite assemblages. We also found strong correlations between the water mite fauna and both conductivity and pH gradient. Our results show that water conductivity is the most important of the examined factors, driving mite-species distribution in peatlands.

## Introduction

Water mites are the most species-rich group of arachnids occurring in standing and running freshwater habitats (about 6,000 species worldwide; Di Sabatino et al. 2002; Smith et al. 2009). Their ecology has been neglected relative to that of most groups of freshwater arthropods. This is due to the poorly studied, variable life cycles, and difficult taxonomy of juvenile and adult stages. Water mite life cycles normally consist of several inactive pupa-like stages, an active living larva, deutonymph and adult stage (Smith et al. 2009). The larvae parasitize various groups of invertebrates (Diptera, Trichoptera, Coleoptera, Plecoptera, Heteroptera, Odonata and Collembola), which enables water mites to disperse and colonize various habitats (Martin 2008). The water mites are excellent water quality indicators in various ecosystems (Biesiadka and Kowalik 1991; Rousch et al. 1997; Di Sabatino et al. 2002; Dohet et al. 2008). Because they are predators as nymphs and adults, water mite species richness may reflect diversity of prey animals, and changes in mite faunal diversity should reflect changes in food web structure caused by physical and/or chemical disturbance as well as their own sensitivity to environmental conditions. As parasites (larvae), water mites are also dependent on the habitat providing the host’s environmental requirements.

The distribution and ecology of water mites in peatlands has been qualitatively and semi-quantitatively studied e.g., in Germany (Schieferdecker 1966), Sweden (Lundblad 1968), Poland (Biesiadka and Kowalik 1991; Cichocka 1995, 1998) and The Netherlands (e.g. Smit and Van der Hammen 2000). Most of the studies concentrated only on presence and abundance of hydrachnidians in peatland habitats without including morphological or chemical aspects of the habitats. Moreover, most studies are aimed at the fauna of bogs, whereas spring fens are rarely studied. This article is the first attempt at defining the relationships between water mites and their environment, in particular the poor-rich gradient in selected Polish peatlands.

Aquatic and semiaquatic microhabitats characterized by a wide range of variation in water levels, conditions, substrates, and vegetation are inhabited by different water mite species (Smith et al. 2009). We have chosen environmental variables such as water depth, dissolved oxygen content, temperature, pH, and conductivity because they are easily and inexpensively measured in the field. Conductivity reflects the mineral richness of water and is often used to show the total concentration of ions (Chytrý et al. 2003). Additionally, conductivity of groundwater positively correlates with calcium content and pH (Gąbka and Lamentowicz 2008; Bourbonniere 2009). Horsák (2006) and Hájek et al. (2006) found no correlation between these parameters, but only when conductivity exceeded 300 μS/cm, as found in extremely calcium-rich fens. Therefore, both conductivity and pH can be used to estimate mineral richness of the water in peatlands. These parameters are also the most temporally stable variables controlling species composition of plant communities (Hájek et al. 2006).

We formulated the following hypotheses: 1) water mites, as organisms sensitive to biotic and abiotic environments, should strongly reflect the different conditions within the microhabitats; 2) different assemblages of water mites should occur along the mineral richness gradient that influences plant assemblages.

The detailed aims of this study are: (a) to identify distinctive microhabitat types in Polish peatlands based on conductivity, pH, temperature, water depth, dissolved oxygen, vegetation, and to determine their species composition; (b) to quantify the most important environmental parameters correlated with variation in water mite assemblages.

## Methods

### Study area characteristic

**Fig. 1:**
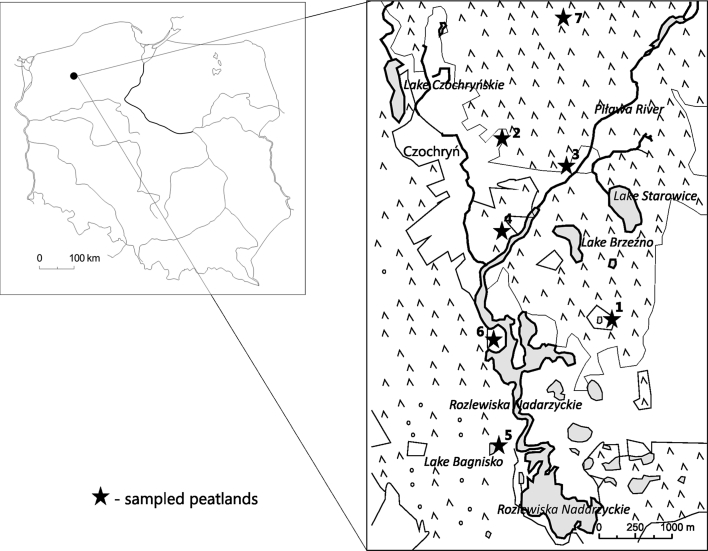
Map of the position of the sampled peatlands within Poland. The *asterisks* and *numbers* represent the individual peatlands: *1* Młyńskie Bagna, *2* Starowice, *3* Starowice Mostek, *4* Torfowisko koło Starowic, *5* Jezioro Bagnisko, *6* unnamed spring fen, *7* Torfowisko nad Piławą

The study site is located in the moraine area of the Western-Pomeranian Lakeland within the Drawsko Lakeland (Fig. 1; Table 1). The seven studied wetlands are situated in coniferous forest areas dominated by *Pinus silvestris* and have kettle hole-shaped basins with steep slopes. We tested a width variety of natural, semi-natural and disturbed peatland habitats, which differed in base richness (poor-rich) and water depth (wet-dry). The oligotrophic-acid conditions enable the development of vegetation typical of ombrotrophic habitats. However, the exploitation of peat in the Starowice and Torfowisko nad Piławą (until 1934) allowed the area to establish vegetation of eutrophic small water bodies. The most natural conditions containing well-developed floating mat margins are represented by Jezioro Bagnisko and Młyńskie Bagna. One of the studied peatlands is a spring fen overgrown by plants such as *Carex paniculata*, *Iris pseudacorus*, and *Equisetum fluviatile*, both of which require waters rich in ions.

**Table 1:** Seven sampled peatlands with geographical coordinates and numbers of samples collected in particular microhabitats in May, July and September 2010

Site names	Longitude	Latitude	Number of samples
Młyńskie Bagna	N:53°30′51′′	E:16°29′44′′	16
Jezioro Bagnisko	N:53°29′56′′	E:16°28′39′′	18
Torfowisko nad Piławą	N:53°32′46′′	E:16°29′19′′	14
Spring fen	N:53°30′43′′	E:16°28′34′′	12
Starowice	N:53°32′2′′	E:16°28′39′′	14
Starowice Mostek	N:53°31′49″	E:16°29′14″	7
Torfowisko koło Starowic	N:53°31′25″	E:16°28′39″	6

### Field sampling and laboratory analysis

We took a total of 71 samples from our studied peatlands between July and September 2010. In order to compare composition of invertebrate assemblages between different seasons we collected 16 additional qualitative samples in May 2010 (Table 1). Each sample had a volume of 10 liters and was collected using a net (mesh size 250 μm) with a triangular frame with a 20 cm edge. Materials collected from the study sites with low water depth (e.g. floating-mat margins, marginal region of the peatland) were taken five times with a 2 l bucket (modified after Lipinski and Kiel 2009; Smith et al. 2009). Vegetation was surveyed on standard plots of 1 m^2^. We used the Braun-Blanquet method for vegetation records to first estimate the species cover in the field, these values were transformed into a point scale according to Van der Maarel (1979).

Each peatland consisted of several microhabitat types. Within the sampled vegetation plots, seven types of microhabitats were arranged from poorest to richest in minerals; they differed in terms of water level and conditions, vegetation and physicochemical parameters. These microhabitats were: (1) pools of poor acid *Sphagnum* bogs; (2) floating-mat margins of poor acid peat bogs (*Carex limosa* and *Rhynchospora alba* communities); (3) hollows in hummock-hollow complexes located in bogs dominated by *Sphagnum fallax;* (4) marginal parts of *Sphagnum* bogs with vegetation habitats richer in minerals (*Juncus effusus*); pools with *Stratiotes aloides* typical of eutrophic waters; (5) spring waters of nutrient-rich spring fen; (6) streams overgrown by *Cardamine amara* and *Lemna minor* located in spring fen; (7) rushes of spring fen with *Equisetetum* *fluviatile*.

We sorted samples in the laboratory under a stereomicroscope. Water mites and other invertebrates were preserved in 70 % ethanol. We identified water mites to species level and other invertebrate taxa to coarser taxonomic levels (e.g. Viets 1936; Kołodziejczyk et al. 1998; Gerecke et al. 2007, 2010). For the potential prey and/or host invertebrates, only presence is given here (see Table 2). All parameters were measured directly in the field in each microhabitat at the same time. We used portable instruments to measure values of temperature, conductivity, pH and dissolved oxygen (ELMERON CX 401). Water depth was measured with a centimeter tape, where the zero level was determined by top of the *Sphagnum* plants.

**Table 2:** Prey and host taxa present in water mite habitats

Mite taxon	Number of specimens	Present prey taxa	Known prey taxa from literature	Present host taxa	Known host taxa from literature
*Hydryphantes placationis* Thon	1	**–**	**–**	**–**	**–**
*H. ruber* (Geer)	3	Culicidae larvae	Culicidae larvae (Mullen 1975)	Culicidae (larvae, pupae)	Culicidae (Piersig 1896–1899)
*Parathyas bruzelii* (Lundblad)	3	**–**	**–**	**–**	**–**
*Zschokkea oblonga* Koenike	2	**–**	**–**	**–**	**–**
*Piersigia intermedia* Williamson	5	**–**	**–**	**–**	**–**
*P. koenikei* K. Viets	1	**–**	**–**	**–**	**–**
*Limnochares aquatica* (L.)	3	Diptera larvae, Ephemeroptera, Cladocera	Chironomidae larvae, wounded animals (mayfly larvae, *Asellus aquaticus*, *Daphnia*) (Böttger 1969)	Heteroptera, Odonata	Gerridae, Hydrometridae, Mesoveliidae, Odonata (Böttger 1969)
*Piona carnea* (Koch)	4	Diptera larvae, Cladocera	Chironomidae larvae, Cladocera (Paterson 1970)**	Diptera	Chironomidae (Kouwets and Davids 1984)
*P. clavicornis* (Müller)	3	–	**–**	**–**	**–**
*P. conglobata* (Koch)	14	Cladocera	*Ceriodaphnia* (Proctor and Pritchard 1989)	Diptera	Chironomidae (Kouwets and Davids 1984)
*T. ensifer* (Koenike)	1	–	**–**	**–**	**–**
*T. latipes* (Müller)	2	Absent	Cladocera, Copepoda, Ostracoda**	**–**	**–**
*T. scaurus* (Koenike)	6	**–**	**–**	**–**	**–**
*Limnesia connata* Koenike	58	–	**–**	–	No host (Van Haaren and Tempelman 2009)
*Lebertia separata* Lunblad	4	–	–	–	–
*Sperchon squamosus* Kramer	6	Chironomidae larvae	Chironomidae larvae Simuliidae larvae***	Chironomidae	Chironomidae (Martin and Stur 2006)
*Hygrobates norvegicus* (Thor)	1	Chironomidae larvae, mites (*Sperchon squamosus*)	Chironomidae larvae, mites (e.g. *Sperchon* sp.)***	Chironomidae	Chironomidae (Martin and Stur 2006)
*Hydrodroma despiciens* (Müller)	20	Odonata larvae, Diptera larvae	Dragonfly eggs (Lanciani 1978), caddisfly eggs (Wiles 1982), Chironomidae eggs (Meyer 1985)	–	**–**
*H. pilosa* Besseling	1	–	–	Trichoptera	Chaoboridae, Chironomidae, Tipulidae, Leptoceridae, Limnephilidae (Wiles 1987)
*Arrenurus bifidicodulus* Piersig	1	**–**	**–**	**–**	**–**
*A. stecki* Koenike	28	–	**–**	Diptera	Culicidae*
*A. perforatus* George	1	–	**–**	**–**	**–**
*A. cylindratus* Piersig	10	Ostracoda	Ostracoda***	–	**–**
*A. globator* (Müller)	1	Copepoda, Ostracoda	Cladocera, Ostracoda, Copepoda**	Diptera	Dytiscidae larvae, Culicidae, Dixidae*
*A. tubulator* (Müller)	8	–	**–**	Diptera	Culicidae, Dixidae*
*A. mediorotundatus* Thor	2	–	**–**	**–**	**–**
*A. bruzelii* Koenike	3	–	**–**	Odonata	Odonata
*A. claviger* Koenike	1	–	**–**	Odonata	Odonata*
*A. cuspidator* (Müller)	1	Cladocera, Ostracoda	Cladocera, Ostracoda (?)**	Odonata	Odonata (Stechmann 1978)*
*A. maculator* (Müller)	6	–	**–**	Odonata	Odonata*
*A. neumani* Piersig	12	Cladocera, Copepoda	Cladocera, Ostracoda, Copepoda**	Odonata	Odonata*
*A. pustulator* (Müller)	1	–	**–**	Host absent	Odonata*

Adults and nymphs are predators, larvae are parasites. Previous records indicated by the literature are not exhaustive. In studied habitats were recorded also Anisitsiellidae indet. (2 larval specimens), Hydryphantidae (1), *Arrenurus* sp. (32), *Piona* sp. (24), *Limnesia* sp. (2) and *Hydryphantes* sp. (1). Records from literature are given in parenthesis

^*^Zawal (2008)

^** ^Böttger (1970)

^***^Martin (2005)

### Numerical analysis

Canonical redundancy analysis (RDA) was used to analyze the most significant factors correlated with water mite species composition. As peatlands are typically habitats poor in water mite species richness and abundances (Viets 1938; Cichocka 1998), it was not surprising that water mites occurred in only 49 of 70 samples. As a result in RDA ordination, pools and temporary habitats were better represented than spring water sites. Statistical analyses were performed using CANOCO software (Ter Braak and Šmilauer 1998).

## Results

### Water mites, their hosts and prey

A total of 275 water mites representing 32 species were identified (Table 2). We collected more than 2,500 specimens of other invertebrate taxa comprising molluscs, leeches, crustaceans, caddisflies, mosquitoes, chironomids, odonates, mayflies, stoneflies, beetles, true bugs, springtails, and other arachnids.

The most abundant water mite species were *Limnesia connata* (58 specimens), *Arrenurus stecki* (28), *Hydrodroma despiciens* (20) and *Piona conglobata* (14). Additionally, we found one species, *Lebertia separata* which is a new record for Polish fauna (Biesiadka 1997, 2008). Moreover, we found *Piersigia intermedia*, *Zschokkea oblonga*, *Sperchon squamosus*, *Hygrobates norvegicus* and *A. pustulator* which are also relatively rare in Europe (Smit and Van der Hammen 2000; Biesiadka 2008). In addition, among the other invertebrate groups there were both water mite prey and host organisms. The characteristics of prey and host taxa of individual water mite species in particular habitats are summarized in Table 2.

### Water mites in relation to environment and vegetation

**Fig. 2:**
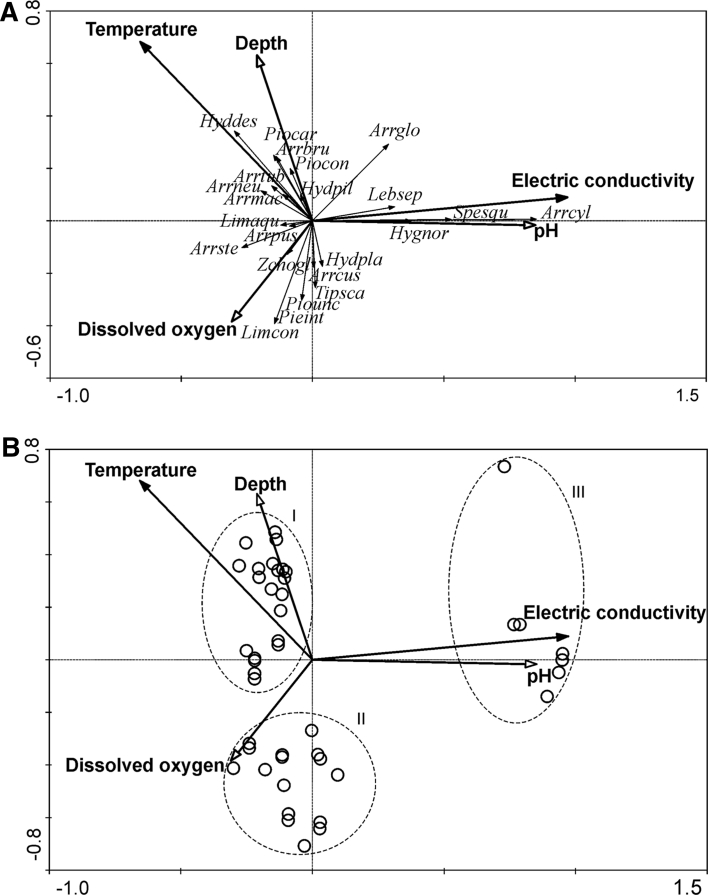
Biplots of canonical redundancy analysis (RDA) for species (**a**) and samples (**b**). Species list: Limcon – *Limnesia connata*, Limaqu – *L. aquatica*, Pieint – *P. intermedia*, Piocar – *Piona carnea*, Piocon – *P. conglobata*, Piounc – *Pionacercus uncinatus*, Tipsca – *T. scaurus*, Hyddes – *H. despiciens*, Hydpil – *H. pilosa*, Lebsep – *L. separata*, Spesqu – *S. squamosus*, Arrbru – *A. bruzelii*, Arrcyl – *A. cylindratus*, Arrcus – *A. cuspidator*, Arrmac – *A. maculator*, Arrneu – *A. neumani*, Arrpus – *A. pustulator*, Arrste – *A. stecki*, Arrglo – *A. globator*, Arrtub – *A. tubulator*, Hygnor – *H. norvegicus*, Hydpla – *H. placationis*, Zchogl – *Z. oblonga*. I – pools, II – temporary habitats, III – ground waters of the spring water fen; the data refer to samples collected in late July and early September 2010

Figure 2 presents two RDA biplots. RDA analysis revealed the main environmental factors correlated with the spatial distribution of water mites (Fig. 2a). The Monte Carlo permutation test determined that the most important factors potentially affecting the water mite fauna were conductivity and temperature (Table 3). The species are mostly grouped along the *y*-axis of the RDA-diagram. On the positive range of the *x*-axis are the spring-inhabiting *S. squamosus*, *A. cylindratus*, *H. norvegicus* and *L. separata*. The lower left cluster represents those species which occur in lower values of pH and conductivity (e.g. *Limnochares aquatica*, *A. pustulator*, *A. stecki*, *Z. oblonga*). Increasing association with higher pH and conductivity was shown for *H. placationis*, *A. cuspidator* and *T. scaurus*. Deeper sampling sites located in pools are inhabited by greater number of species such as *H. despiciens*, *H. pilosa*, *Piona carnea*, *Arrenurus tubulator*, *A. neumani*, *A. maculator* and *A. bruzelii*.

**Table 3:** Results of the forward selection of environmental parameters (Monte Carlo permutation test in CCA, *P* < 0.05 are statistically significant and given in bold)

Parameter	All sites		
	λ	F	*P*
Electric conductivity	0.10	4.94	**0.001**
Temperature	0.05	2.85	**0.003**
Depth	0.02	1.30	0.201
pH	0.03	1.33	0.199
Dissolved oxygen	0.01	0.69	0.723

Three groups of water mite species of habitats with various environmental conditions were distinguished (Fig. 2a): (1) species typical of ground waters, (2) species of pools and (3) species typical of temporary habitats. Group (1) was represented by spring and stream living water mites adapted to a very narrow range of environmental conditions such as crenobiont *H. norvegicus* or *L. separata* (Gerecke et al. 2005, 2010). The main factors governing species composition of these habitats were conductivity and pH. In the stagnant part of the spring fen with higher maxima of temperature *Arrenurus globator* was found, a species present in various kinds of lentic waters (Smit and Van der Hammen 2000). The highest values of water depth and acidic conditions determined species composition of pools. Composition of group 2 consisted of *H.* *despiciens*, *H. pilosa, Piona conglobata, P. carnea, Arrenurus bruzelii, A. neumani, A. pustulator, A. maculator, A. tubulator* and *L. aquatica*. We found there also *A. claviger, L. connata* and *P. clavicornis* in the springtime. The most important abiotic factors influencing representatives of the 3rd group were the highest values of dissolved oxygen in connection with a very low water depth. The third group consists of *P. intermedia, Limnesia connata, Z. oblonga, Arrenurus stecki*, *T. scaurus, H. placationis, A. cuspidator* and *Pionacercus uncinatus*. We recorded in hummock-hollow complex with strong acidic conditions and very low conductivity *Z. oblonga* in the springtime. In the marginal parts of the peatlands with higher values of conductivity and pH occurred in the spring time *Tiphys ensifer, T. latipes, Parathyas bruzelli, Hydryphantes ruber, Arrenurus mediorotundatus, A. bifidicodulus* and *A. perforatus.*

### Environmental variables and vegetation

The chemical parameters measured in summer varied greatly in regards to our results for all sites taken together. Values of pH (4.10–7.60), electric conductivity (22–465 μS/cm), temperature (9.1–28.8 °C) and dissolved oxygen (4.2–112.0 mg O_2_^−1^) differed strongly among and within the studied microhabitats (Table 4). The values of parameters in the spring oscillated by: 3.04–7.07 (for pH), 8.0–16.7 °C (for temperature), 18–67 μS/cm (for electric conductivity), and 1.3–6.6 mg O_2_/l (for oxygen content). Nevertheless they are more stable in groundwater-influenced microhabitats than in those with low water levels and corresponding stronger changes in temperatures etc.

**Table 4:** Physicochemical parameters of the sites sampled in this study. The habitats are classified according to the RDA analysis; the data refer to samples collected in July–September 2010

	Pools (n = 25)			Temporary habitats (n = 17)			Ground waters (n = 10)		
	Min.	Max.	Mean	Min.	Max.	Mean	Min.	Max.	Mean
pH	4.4	5.8	5.3	4.1	6.5	5.2	5.7	7.6	7.2
Conductivity (μS/cm)	23	32	29	28	103	51	341	465	373
Temperature **(**°C)	19.1	28.8	27.4	12.4	26.6	16.3	9.1	18.5	11.6
O_2_ dissolved (mg/l)	5	7	7	4	11	8	4	7	5
Water depth (cm)	30	60	47	0	40	12	5	40	22

Based on the RDA analysis, the seven microhabitat types were clustered in three groups (Fig. 2b): permanent pools (areas with poorly developed vegetation belts, pools with an abundant growth of macrophytes), temporary habitats (floating-mat margins, marginal part of peatland, hummock hollow complex), groundwater influenced habitats of a spring fen (limnocrene, helocrenes, streams, rushes). The permanent pools were characterized by the highest water depth and the lowest conductivity values; the largest area was occupied by vegetation typical of oligo—(mesotrophic)—acid conditions such as *Sphagnum fallax*, *Carex limosa*, *Rhynchospora alba*, *Menyanthes trifoliata* and *Utricularia minor*. The temporary habitats are well oxygenated semiaquatic habitats such as floating-mat margins, hollows and marginal parts of peatlands. The quaking bogs were covered mainly by oligotrophic-acidic vegetation: *Sphagnum* mosses (mainly *Sphagnum fallax*), *Rhynhospora alba*, *Scheuchzeria palustris*, *Drosera rotundifolia, Drosera anglica, Oxycoccus palustris, Peucedanum palustre, Hydrocotyle vulgaris, Utricularia minor* and *Potentilla palustris.* The marginal parts of peat bogs and hollows were overgrown by plants, which are typical for waters richer in ions (*Phragmites australis*, *Typha latifolia, Juncus effusus*, *Calla palustris,* *Calliergonella cuspidata*). The springs plants located in the spring fen consist of *Lemna minor, Carex pseudocyperus, Berula erecta* and *Carex paniculata*. The streams were covered by *Carex paniculata, Lemna minor, Cardamine amara, Veronica buccabunga, Iris pseudacorus* and *Mentha aquatica*. The rushes of the spring fen were overgrown by *Equisetum fluviatile, Manna aquatica, Rumex hydrolapathum, Cardamina amara, Hydrocharis morsus*-*ranae, Lemna minor* and *Cicuta virosa*. These groundwater influenced habitats had the lowest values of temperature and the highest values of electric conductivity and pH compared to the other sites.

Most samples on the RDA-diagram constitute a cloud of dots extended along the y-axis (Fig. 2b). The left part of the diagram groups oligotrophic-acidic microhabitats. Along the vector of water depth cluster samples collected in pools. In the lower left part of the RDA-diagram temporary habitats group such as quaking bogs, hollows and marginal parts of peatlands which positively correlate with dissolved oxygen. On the right edge sample sites cluster are strongly correlated with conductivity and pH, parameters which were located in spring fen. There is also a strong positive correlation between pH and conductivity. The vectors of the water depth and dissolved oxygen are located opposite what indicates a negative correlation between these parameters. This may be associated with intensive processes of photosynthesis occurring in shallow peatland habitats. A negative correlation exists also between dissolved oxygen and electric conductivity. An isolated data point at the right upper edge represents rushes under influence of ground waters.

The peat bog microhabitats sampled in springtime were marginal parts of peatlands and hollows of the hummock-hollow complex which dry out in summer months. They were covered by *Sphagnum* *fallax* and plants typical for waters richer in ions: *Juncus effusus*, *Carex palustris*, *Equisetum fluviatile*, *Potentilla palustris* and *Menyanthes trifoliata*. The hollows were characterized by lower water level and values of pH as well as higher concentrations of ions and oxygen.

## Discussion

### Relation between water mites and environmental conditions

There are no studies focused on the role of selected abiotic parameters on species composition of water mites in peatlands, though there is strong evidence for some influence of single factors on explaining patterns of mites in different habitats.

Smit and Van der Hammen (1992) who investigated coastal dune areas in the Netherlands and northwestern France studied the influence of physicochemical parameters on species composition of water mite fauna. They mentioned the dimension, amount of water vegetation, pH and nutrient concentration as the most important environmental variables affecting species composition. On a larger geographic level, Goldschmidt (2004) showed for various freshwater habitats in Costa Rica that water chemistry exhibits less impact on the differentiation of water mite assemblages than habitat type, elevation, temperature and velocity. However, he emphasized the need for further detailed studies at the species level concentrating on microhabitats as we did in our study.

Here, we described the water mites of peatland microhabitats in relation to water depth, temperature, electric conductivity, pH, dissolved oxygen, vegetation, and other invertebrates. The distinguished microhabitats in this study which differed in terms of water level, physicochemical conditions and vegetation were inhabited by water mites which typically occupy narrow ranges of environmental conditions (Smit and Van der Hammen 2000). It is well known that acidic waters of *Sphagnum* peatlands are poor in inorganic ions (Ca^2+^, Cl^−^, SO_4_^2−^), which may be expressed in values of conductivity (Wheeler and Proctor 2000; Horsák 2006). Here, we found differences in water mite assemblages in various peatland habitats which, may be influenced by ionic richness. Similar observations were already made for water mites in other habitats. Böttger (1980) studied streams in Guatemala with different conductivities and alkalinities which differed significantly in their water mite assemblages. In our study we also observed a correlation between mineral richness and the spring-living *L. separata,* *H. norvegicus,* *S. squamosus* and *A. cylindratus*. Also the pattern of local species composition along the gradient of conductivity is similar to that of water pH. The importance of mineral richness on the distribution of water mite species in Central Europe was emphasized by Schwoerbel (1961). There have been no statistical studies on an impact of conductivity and calcium content on the composition and structure of water mite assemblages in various wetlands.

Most water mite species occurring in bogs are likely not strictly dependent on acidic conditions but rather more resistant than other species. Such stress-tolerant species as *P. conglobata* and *A. bruzelii* are known from a wide range of stagnant aquatic habitats (Kreuzer 1940; Schieferdecker 1966; Smit and Van der Hammen 2000). However, there are also data from the literature suggesting that such water types are inhabited by water mites typical of mineral-poor, acidic environments. Previous data on the preference of *Piona carnea* for acidic waters of pools given in the literature seem to be incorrect. Various authors report the presence of *P. carnea* in acidic dystrophic waters (Biesiadka and Kowalik 1991), in nutrient-rich fish ponds (Biesiadka 2008) and in coastal dunes (Smit and Van der Hammen 1992). This species may have the ability to tolerate environmental conditions where the physicochemical factors are too extreme for its rivals and predators. It is also possible that there is more than one specialized species (H. Proctor pers. comm.). The well known distribution of *L. aquatica* in many habitats other than bogs has probably little in common with specific properties of water but rather with large amount of peat deposits (Viets 1938; Schieferdecker 1966). It can also relate to interactions with other mite species, optimal food supply and/or predation on *L. aquatica*. However, *P. intermedia*, *Z. oblonga* and *A. stecki* seem to prefer semiaquatic habitats with *Sphagnum* and thus support the similar findings of Schieferdecker (1966) and Van Maanen et al. (1997). Moreover, there are some species (*Arrenurus neumani*, *A. pustulator*) which seem to occur in acidic waters low in nutrients, and with an abundant growth of macrophytes (Biesiadka and Kowalik 1991; Cichocka 1998; Smit and Van der Hammen 2000).

### Why are vegetation and other invertebrates of great importance for water mites?

There are a few reasons why vegetation may explain much wider variability than mere water chemistry. Firstly climate and geomorphology affect plant communities building potential microhabitats. Secondly, aquatic and subaquatic plants create substrate using different phases of the linked life cycles of water mites and their hosts. For example many water mites lay eggs and transform from deutonymphs to tritonymphs among aquatic mosses and macrophytes (Smith et al. 2009). Additionally, the mosses and plants offer suitable conditions for hosts thus playing a large role in the dispersion and colonization of new habitats by water mites (Martin 2008). The description of the vegetation structure and gradient analysis presented in this work will be helpful in understanding the mechanisms of the general dispersion of hydrachnidians in peatlands.

Water mites have few enemies and thus, predation does not play an important role in the life of water mites (Smith et al. 2009). Most water mite species have parasitic larvae which enables their dispersal. For peat bogs Viets (1938) speculated that the acidic pools in the center of the peat bogs were arbitrarily invaded by hosts from the surroundings. Perhaps, the species are not able to reproduce in these “new” habitats. The absence of host taxa in the present study (e.g. for *A. pustulator*; see Table 2) may be caused by such a phenomenon. Mitchell (1964) observed dispersal of water mites from adjacent habitats and he distinguished between “resident” and “immigrant” species of the studied genus *Arrenurus*. These laboratory and field studies illustrated that the tolerance of the “predatory phase” of *Arrenurus* (from the subgenus *Arrenurus*) is much broader than that of the parasitic phase. In his opinion, failure in reproduction limits the success of these species. One-fourth of the studied species was unable to reproduce because of unfavorable conditions for the parasitic larva. These conditions were absence of the host, behavioral features of larvae and some environmental factors.

We found the hosts with a high dispersal capacity (Odonata) in various parts of the studied peatlands including centrally located pools. However, the spread of particular species can not always be explained by possession of the parasitic larvae (Table 2). Another interesting species is *L. connata* which was one of the most successful colonizers of various peatland habitats but seems to have lost the parasitic stage (Van Haaren and Tempelman 2009). The majority of specimens of *L. connata* inhabited temporary marginal parts of peatlands, hollows of the hummock-hollow complex and shallow pools. However, this species occurs also in permanent pools located in central parts of the peat bogs.

## Conclusion

Peatlands are a mosaic of aquatic and semiaquatic habitats differing in water level, chemistry, vegetation structure and shadowing. Thus, they are suitable areas for studies concentrating on relationships between water mites and their environmental correlates but also to the study on their biotic interactions with their hosts and prey organisms.
